# Association between acupuncture and IVF outcomes in women with poor ovarian response: a retrospective cohort study

**DOI:** 10.3389/fcell.2026.1756473

**Published:** 2026-07-08

**Authors:** Jia Liu, Ran Ji, Xiaoyan Zheng, Jingwen Zhang, Han Yang, Liying Liu, Qingran Kong, Xue Xiao, Xingyu Lv, Jie Yang, Xin Sun

**Affiliations:** 1 West China Second University Hospital, Sichuan University/West China Women’s and Children’s Hospital, Chengdu, China; 2 Acupuncture and Tuina School, Chengdu University of Traditional Chinese Medicine, Chengdu, China; 3 Division of Internal Medicine, Institute of Integrated Traditional Chinese and Western Medicine, West China Hospital, Sichuan University, Chengdu, China; 4 Department of Gynecology and Obstetrics, West China Second University Hospital, Sichuan University, Chengdu, China; 5 Key Laboratory of Birth Defects and Related Diseases of Women and Children (Sichuan University), Ministry of Education, West China Second Hospital, Sichuan University, Chengdu, China; 6 Tianfu Jincheng Laboratory, Chengdu, China; 7 Laboratory of Stem Cell and Embryo Development, West China Second Hospital, Sichuan University, Chengdu, China; 8 Reproductive Center, Sichuan Jinxin Xi’nan Women’s and Children’s Hospital, Chengdu, China; 9 West China Hospital, Sichuan University, Chengdu, China

**Keywords:** acupuncture, assisted reproductive technology, cohort study, *in vitro* fertilization, oocyte, poor ovarian response

## Abstract

**Introduction:**

Poor ovarian response (POR) is a major barrier to *in vitro* fertilization (IVF) success. We assessed whether adjunctive acupuncture is associated with improved IVF outcomes among women with POR.

**Methods:**

We conducted a retrospective cohort study of women with POR undergoing IVF at Sichuan Jinxin Xi’nan Women’s and Children’s Hospital (1 January 2020–31 October 2024). Patients selected acupuncture (typically three sessions per week from ovarian stimulation through the day of embryo transfer) or no acupuncture in either acupuncture group or control group. The primary endpoint was the number of oocytes retrieved. The secondary endpoints included the clinical pregnancy rate (CPR) and live birth rate (LBR). Subgroup analyses were conducted according to the acupuncture dosage (low-dose group: ≤4 sessions, high-dose group: >4 sessions).

**Results:**

A total of 2,133 women with POR were included (887 in the acupuncture group and 1,246 in the control group). The baseline demographic and clinical characteristics were comparable between the groups except for follicle-stimulating hormone (FSH) and anti-Müllerian hormone (AMH), and the previous failure cycles, which were adjusted as covariates in multivariable logistic regression. Regarding the primary endpoint, the higher number of retrieved oocytes (median [Q1, Q3]) in the acupuncture group (4.0 [3.0, 7.0] versus 3.0 [1.0, 4.0], Standardized Mean Difference (SMD) = 1.7, 95% confidence interval (CI) [1.53–1.95], *P* < 0.001, acupuncture group vs. control group). Higher CPR was also obtained in the acupuncture group (20.74% vs. 15.73%, adjusted odds ratio (OR) = 1.2, 95% CI [1.2–1.3], *P* = 0.037). However, the difference in LBR between the two groups did not reach statistical significance (13.8% vs. 13.9%, *P* = 0.059, adjusted OR, 1.0, 95% CI [0.9–1.1], acupuncture group vs. control group). Subgroup analyses indicated that high-dose acupuncture resulted in significantly higher oocytes retrieved (5.00 [3.00, 7.00] vs. 4.00 [3.00, 6.00], adjusted SMD = 0.5, 95% CI [0.3–0.7] *P* < 0.001).

**Conclusion:**

In this single-center retrospective cohort, acupuncture was associated with an increased number of retrieved oocytes among women with POR undergoing IVF. Causal inference is limited; randomized trials using standardized acupuncture protocols are warranted.

## 1 Introduction

POR is a persistent challenge in assisted reproductive technology (ART), affecting 9%–24% of women undergoing COH and substantially reducing IVF success rates (Zhang et al., 2020; Pandian et al., 2010; Polyzos and Devroey, 2011; Rasool and Shah, 2017). Current management strategies, including optimized ovulation induction and adjunctive therapies like growth hormones, are heterogeneous and lack robust, generalizable evidence, highlighting a need for safe, accessible adjuncts (Giannelou et al., 2020; Pantou et al., 2021; Merviel et al., 2015).

Acupuncture, a method of Chinese medicine that includes inserting needles into specific points of the body, has acquired more and more popularity in the field of reproductive medicine as its side effects are minimal (Witt et al., 2009). Research indicates the possibility of using acupuncture as a means to enhance ovarian responsiveness through manipulation of the hypothalamic-pituitary-ovarian (HPO) axis, stimulation of ovarian blood flow, and reduction of oxidative stress (Feng et al., 2021). Still, the effectiveness of acupuncture in POR remains inconclusive, and the safety and efficacy of this method require additional real-life data (Wang et al., 2023). We hypothesized that adjunctive acupuncture would be associated with an increased number of oocytes retrieved and improved pregnancy outcomes, and used real-life data to investigate the relationship between acupuncture and a better outcome of *in vitro* fertilisation (IVF) in women who have POR.

## 2 Materials and methods

### 2.1 Study design and setting

**FIGURE 1:**
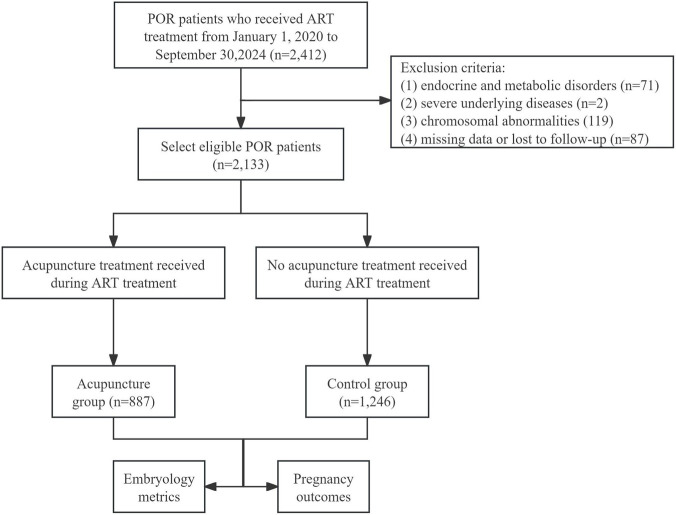
POR: poor ovarian response; ART: assisted reproductive technology.

We performed a retrospective cohort study at Sichuan Jinxin Xi’nan Women’s and Children’s Hospital, including all eligible IVF cycles between 1 January 2020, and 31 October 2024. The institutional review board approved the study (IRB-KY-2021–001). Informed consent was not required because this research was a secondary data analysis. In this study, a retrospective design was used hence no informed consent was needed. The research followed the suggested protocols of observational research that had been conducted based on routinely collected health data (Figure 1) (Supplementary Table 1).

### 2.2 Participants

Eligibility required meeting the Bologna criteria for POR (Ferraretti et al., 2011) and age 21–40 years. Ovarian stimulation was administered based on the gonadotropin-releasing hormone (GnRH) antagonist regimen (Chen et al., 2022). We excluded individuals with uncontrolled endocrine or metabolic disease (e.g., thyroid dysfunction, diabetes, polycystic ovary syndrome), severe systemic illness (e.g., cardiovascular/cerebrovascular disease, hepatic/renal insufficiency, malignancy), chromosomal abnormalities (e.g., Turner syndrome, balanced translocations), ≥20% missing data, or loss to follow-up. Patients who had received acupuncture treatment for any indication within the 3 months preceding the IVF cycle were excluded to minimize potential selection bias.

### 2.3 Exposure (acupuncture protocol)

Patients self-selected into an acupuncture group or a control group. For fresh embryo transfer, acupuncture was administered during the controlled ovarian hyperstimulation (COH) and embryo transfer stages. For frozen embryo transfer, acupuncture was performed during the endometrial preparation phase until embryo transfer. Acupuncture sessions were typically conducted every other day for 30 min, with the total number of sessions determined by the patient’s preference and individual circumstances. Two alternating point prescriptions were used: set 1 (GV20 (Baihui), CV12 (Zhongwan), ST25 (Tianshu), CV6 (Qihai), CV4 (Guanyuan), GB26 (Daimai), KI12 (Dahe), EX-CA1 (Zigong), SP10 (Xuehai), ST36 (Zusanli), and LR3 (Taichong)) and set 2 (GV20 (Baihui), BL23 (Shenshu), BL32 (Ciliao), SP6(Sanyinjiao), KI3(Taixi)). Detailed locations and needling parameters are provided in Supplementary Table 2. Only one set of acupoints is used per treatment session. The first treatment used the first prescription. After disinfection, needles were inserted at a 90-degree angle to a depth of 20–30 mm, with manual manipulation including rotation, lifting, and thrusting to elicit the deqi sensation (manifested as soreness, numbness, distention, or a feeling of heaviness). All acupuncture procedures were performed by licensed practitioners with at least 3 years of clinical experience.

### 2.4 Outcomes

The primary outcome was the number of retrieved oocytes. The secondary outcomes included the number of metaphase II (MII) oocytes and two-pronuclei (2PN) zygotes, as well as the type of embryo, all of which play a crucial role in evaluating oocyte quality, embryo developmental potential, and the likelihood of successful implantation (Chamayou, 2022; González et al., 2023). The pregnancy outcomes included the biochemical pregnancy rate (BPR) (biochemical pregnancy cycles/transfer cycles), clinical pregnancy rate (CPR) (ultrasound-confirmed gestational sac/transfer cycles), and the live birth rate (LBR), defined as delivery at ≥22 weeks’ gestation with signs of life (Zegers-Hochschild et al., 2017).

### 2.5 Sample size

The sample size was calculated based on the primary outcome of retrieved oocytes. Using a mean difference of 1.02 (95% CI: 0.72–1.32) from prior POR studies (Wang et al., 2023), a standard deviation of 3.0, α = 0.05, and 90% power, 182 participants per group were initially required. After adjusting for high heterogeneity (I^2^ = 83%) with a 1.5-fold random-effects model multiplier and 20% dropout rate, the final sample size was determined as 341 per group. It is important to acknowledge that, as a retrospective study, the analytical sample size was constrained by data availability. To address missing baseline data, we employed multiple imputation with 5 imputations and 50 iterations per imputation, with one randomly selected imputed dataset used for the primary analysis.

### 2.6 Statistical analysis

Normally distributed continuous variables were expressed as mean ± SD and analyzed using t-tests, whereas non-normally distributed data were reported as median (IQR) and compared via the Mann–Whitney U test. Categorical variables were assessed with the χ^2^ test or Fisher’s exact test as appropriate. Multivariable logistic regression was used to estimate aORs and 95% CIs for BPR, CPR and LBR, with adjustment for pre-defined baseline covariates: female age, baseline FSH, baseline AMH, and prior IVF failure cycles. Multivariable linear regression with identical covariates was used for continuous outcomes including oocyte yield. Pre-planned subgroup analyses were stratified by acupuncture sessions (≤4 vs. >4), maternal age (<35 vs. ≥35 years), AMH (<1.2 vs. ≥1.2 ng/mL) and AFC (<5 vs. ≥5) (Grisendi et al., 2019; Tian et al., 2021). A two-sided P < 0.05 was considered statistically significant. For the pre-planned subgroup analyses, given their exploratory nature, no adjustment for multiple comparisons was applied. Results from these analyses should be interpreted as hypothesis-generating. All reported *P*-values are nominal.

## 3 Results

### 3.1 Participant characteristics

We included 2133 women with POR (887 were in the acupuncture group and 1246 were in the control group). As shown in Table 1, demographic characteristics (including ethnicity, education level, body mass index (BMI)), infertility history (infertility duration and infertility diagnosis), and the baseline ovarian function (the levels of LH, progesterone, and estradiol) were comparable between groups. However, the levels of FSH (*P* < 0.001), AMH (*P* < 0.001), and previous IVF cycles (*P* < 0.001) were imbalanced between groups. Patients in the acupuncture group had lower median FSH (7.8 [6.0, 10.0] vs. 8.5 [6.6, 10.8], *P* < 0.001, acupuncture group vs. control group) and higher median AMH levels (0.9 [0.5, 1.4] vs. 0.8 [0.4, 1.3], *P* < 0.001, acupuncture group vs. control group), and a larger proportion had not undergone prior IVF cycles (43.97% vs. 33.79%, *P* < 0.001, acupuncture group vs. control group).

**TABLE 1:** Characteristics of the patients.

Characteristics	Acupuncture group (n = 887)	Control group (n = 1246)	p-value
Age (y) n (%)	​	​	<0.001
≤30	143 (16.1)	201 (16.1)	​
31–35	368 (41.5)	401 (32.2)	​
≥36	376 (42.4)	644 (51.7)	​
Ethnicity n (%)	​	​	0.059
Han	793 (89.4)	1145 (91.9)	​
Minority	94 (10.60)	101 (8.1)	​
Education level n (%)	​	​	0.189
Low	533 (60.1)	801 (64.3)	​
Medium	160 (18.0)	194 (15.6)	​
High	194 (21.9)	251 (20.2)	​
BMI (kg/m2) median (IQR)	22.07 (4.2)	22.03 (4.4)	0.734
Sex hormones median (IQR)			
LH (mIU/mL)	3.3 [2.3, 4.6]	3.0 [0.6, 5.1]	0.556
FSH (mIU/mL)	7.8 [6.0, 10.0]	8.5 [6.6, 10.8]	<0.001
P (ng/mL)	0.5 [0.3, 0.7]	0.4 [0.3, 0.9]	0.544
E2 (pg/mL)	32.8 [25.0, 45.6]	34.0 [23.0, 46.3]	0.110
AMH (ng/mL)	0.9 [0.5, 1.4]	0.8 [0.4, 1.3]	<0.001
Infertility duration (y) median (IQR)	3.0 [1.0, 5.0]	3.0 [2.0,6.0]	0.781
Infertility factor n (%)	​	​	0.810
Tubal factor	348 (39.3)	375 (30.1)	​
Ovulatory disorder	188 (21.2)	372 (29.9)	​
Mixed factors	258 (29.1)	374 (30.0)	​
Other female causes	74 (8.3)	113 (9.1)	​
Endometriosis	14 (1.9)	12 (1.0)	​
Previous IVF cycles (n%)	​	​	<0.001
0	390 (44.0)	421 (33.8)	​
1	202 (22.8)	337 (27.1)	​
2	160 (18.0)	238 (26.8)	​
≥3	135 (15.2)	250 (28.2)	​

BMI: body mass index; IQR, interquartile range; LH: luteinizing hormone; FSH: follicle-stimulating hormone; P: progesterone; E2: estradiol; AMH: anti-Müllerian hormone; IVF, *in vitro* fetilization.

### 3.2 Embryology metrics and pregnancy outcomes

For the primary endpoint, the acupuncture group had significantly more oocytes retrieved than the control group (median [Q1, Q3], 4.0 [3.0, 7.0] versus 3.0 [1.0, 4.0], SMD = 1.7, 95% CI [1.5–1.9], *P* < 0.001). Similarly, the number of MII oocytes (median [Q1, Q3], 4.0 [3.0, 6.0] vs. 2.0 (1.0, 4.0), SMD = 0.3, 95% CI [0.3–0.4], *P* < 0.001, acupuncture group vs. control group), the number of 2PN oocytes (median [Q1, Q3], 3.0 [2.0, 5.0] vs. 2.0 [1.0, 3.0], SMD = 0.3, 95% CI [0.2–0.3], *P* < 0.001, acupuncture group vs. control group), the number of blastocysts (median [Q1, Q3], 1.0 [0.0, 2.0] vs. 0.0 [0.0, 1.0], SMD = 0.8, 95% CI [0.7–0.9], *P* < 0.001, acupuncture group vs. control group) were significantly increased in the acupuncture group than the control group. Additionally, the acupuncture group showed a lower cycle cancellation rate compared to the control group (9.9% vs 24.6%, OR = 0.3, 95% CI [0.3–0.4], *P* < 0.001).

For the pregnancy outcomes, the acupuncture group had higher CPR (20.74% vs 15.73%, OR = 1.4, 95% CI [1.1–1.8], *P* = 0.003) compared to the control group, but did not remain statistically significant after adjustment (adjusted OR = 1.2, 95% CI [1.2–1.3], *P* = 0.037). Furthermore, no statistically significant intergroup differences were observed in the BPR and LBR (26.04% vs 20.79%, OR = 1.3, 95% CI [1.2–1.6], *P* = 0.005, adjusted OR = 1.4, 95% CI [0.9–2.2] and 13.8% vs. 13.9%, OR = 1.0, 95% CI [0.8–1.3], *P* = 0.059, adjusted OR = 1.1, 95% CI [0.9–1.2], respectively). Details are shown in Table 2.

**TABLE 2:** Embryology metrics and pregnancy outcomes.

Characteristics (median [IQR]/n [%])	Acupuncture group (n = 887)	Control group (n = 1246)	*P-value*	Crude OR/SMD (95% CI)	Adjusted *P*	Adjusted OR/SMD (95% CI)
Oocytes retrieved	4.0 (3.0, 7.0)	3.0 (1.0, 4.0)	<0.001	1.7 (1.5–1.9)	<0.001	1.3 (1.0–1.6)
MII oocytes	4.0 (3.0, 6.0)	2.0 (1.0, 4.0)	<0.001	0.3 (0.3–0.4)	<0.001	1.3 (1.0–1.5)
2PN	3.0 (2.0, 5.0)	2.0 (1.0, 3.0)	<0.001	0.3 (0.2–0.3)	<0.001	1.2 (1.0–1.5)
Blastocysts	1.0 (0.0, 2.0)	0.0 (0.0, 1.0)	<0.001	0.8 (0.7–0.9)	<0.001	0.9 (0.7–0.9)
Cycle cancellation rates	88 (9.9)	307 (24.6)	<0.001	0.3 (0.3–0.4)	<0.001	0.6 (0.5–0.7)
BPR	231 (26.0)	259 (20.8)	0.005	1.3 (1.2–1.6)	1.208	1.4 (0.9–2.2)
CPR	184 (20.74)	196 (15.73)	0.003	1.4 (1.1–1.8)	0.037	1.2 (1.2–1.3)
LBR	124 (13.8)	173 (13.9)	0.059	1.0 (0.8–1.3)	0.175	1.1 (0.9–1.2)

OR, adjusted odds ratio; CI, confidence interval; SMD, standardized mean difference; MII, metaphase II; 2PN, two pronucleus; BPR, biochemical pregnancy rate; CPR, clinical pregnancy rate; LBR, live birth rate.

### 3.3 Subgroup analyses

Subgroup analyses consistently showed that, for all predefined strata, the acupuncture group retrieved a significantly greater number of oocytes than the control group. When looking at age - based subgroups, the number of oocytes retrieved was significantly higher in the acupuncture group than in the control group, in both women <35 years (4.0 [3.0, 7.0] vs. 3.0 [2.0, 4.0], adjusted SMD = 0.6, 95% CI [0.5–0.7], acupuncture group vs. control group) and ≥35 years (4.0 [3.0, 6.0] vs. 3.0 [1.0, 4.0], adjusted SMD = 0.6, 95% CI [0.4–0.7], acupuncture group vs. control group). The beneficial effect of acupuncture was also consistent across subgroups stratified by AMH and AFC. The acupuncture group retrieved a significantly higher number of oocytes than the control group, irrespective of AMH<1.2 (4.0 [3.0, 6.0] vs. 3.0 [1.0, 4.0], adjusted SMD = 0.7, 95% CI [0.6–0.9]) or AMH≥1.2 (5.0 [3.0, 7.0] vs. 3.0 [2.0, 5.0], adjusted SMD = 0.6, 95% CI [0.4–0.7]), or AFC<5 (4.0 [3.0, 6.0] vs. 3.0 [1.0, 4.0], adjusted SMD = 0.5, 95% CI [0.4–0.7]) or AFC≥5 (4.0 [3.0, 7.0] vs. 3.0 [1.0, 4.0], adjusted SMD = 0.6, 95% CI [0.5–0.8]). A significant dose-response relationship was identified, with a higher number of oocytes retrieved in patients receiving >4 acupuncture sessions compared to those receiving ≤4 sessions (5.0 [3.0, 7.0] vs. 4.0 [3.0, 6.0], adjusted SMD = 0.5, 95% CI [0.3–0.7], dose >4 sessions vs. dose ≤4 sessions). Details are shown in Table 3.

**TABLE 3:** Subgroup analysis.

Characteristics	Acupuncture group (n = 887)	Control group (n = 1246)	*P*-value	Crude SMD (95% CI)	Adjusted *P*-value	Adjusted SMD (95% CI)
Overall oocytes retrieved						
Age						
<35 (n = 511)	4.0 (3.0, 7.0)	3.0 (2.0, 4.0)	<0.001	0.7 (0.5–0.8)	<0.001	0.6 (0.5–0.7)
≥35 (n = 376)	4.0 (3.0, 6.0)	3.0 (1.0, 4.0)	<0.001	0.7 (0.6–0.8)	<0.001	0.6 (0.4–0.7)
AMH level						
<1.2 (n = 579)	4.0 (3.0, 6.0)	3.0 (1.0, 4.0)	<0.001	0.7 (0.6–0.8)	<0.001	0.7 (0.6–1.4)
≥1.2 (n = 308)	5.0 (3.0, 7.0)	3.0 (2.0, 5.0)	<0.001	0.6 (0.4–0.7)	<0.001	0.6 (0.4–0.7)
AFC number						
<7 (n = 462)	4.0 (3.0, 6.0)	3.0 (1.0, 4.0)	<0.001	0.6 (0.5–0.7)	<0.001	0.5 (0.4–0.7)
≥7 (n = 425)	4.0 (3.0, 7.0)	3.0 (1.0, 4.0)	<0.001	0.5 (0.4–0.7)	<0.001	0.6 (0.5–0.8)
Acupuncture dose						
≤4 (n = 630)	4.0 (3.0, 6.0)	–	<0.001	0.5 (0.3–0.6)	<0.001	0.5 (0.3–0.7)
>4 (n = 257)	5.0 (3.0, 7.0)	–				

SMD, standardized mean difference; CI, confidence interval; AMH, anti-Müllerian hormone; AFC, antral follicle counting.

## 4 Discussion

This retrospective cohort study involved 2,133 women with POR and explored the potential effects of adjunctive acupuncture during IVF. The findings showed that as an adjunctive therapy, acupuncture demonstrates potential to increase the success rates in IVF. Women who underwent acupuncture treatment experienced a high number of oocytes retrieved compared to those who did not receive acupuncture. Such findings indicate that acupuncture could be a contributing factor to the improvement of ovarian functioning, which subsequently results in a better ovarian response to stimulation, which could also result in better outcomes in women with POR subjected to IVF. Despite the above advantages of acupuncture in enhancing ovarian response, the current study needs additional research to validate the claims, as well as to elaborate on the potential mechanism acupuncture might be using to promote its effect. To gain a better understanding of how acupuncture can be inserted into the IVF treatment plan of POR women, it is necessary to conduct more high-quality randomized controlled trials.

Previous studies have demonstrated that POR is prevalent among infertile women, affecting approximately 1 in 10 patients and up to 1 in 3 in certain populations (Jenkins et al., 1991; Papathanasiou et al., 2016; Surrey and Schoolcraft, 2000). The rising prevalence of POR underscores an urgent demand for effective clinical management. Patients with POR typically exhibit elevated basal FSH, reduced AMH, and diminished ovarian reserve, which collectively contribute to suboptimal responses to ovarian stimulation, fewer retrieved oocytes, and impaired fertilization and embryo development, ultimately compromising IVF prognosis (Tarlatzis et al., 2003). Current POR management mainly aims to enhance ovarian responsiveness and fertility outcomes, with available strategies including optimized ovulation induction protocols and various adjuvant interventions such as androgens, growth hormones, mitochondrial activators, and platelet-rich plasma. Nevertheless, these therapies exhibit substantial heterogeneity in efficacy, and none has demonstrated consistent effectiveness across all POR patients (Farimani et al., 2021; Orvieto, 2022; Cakiroglu et al., 2022; Xu et al., 2018). Given the inherent limitations and variable performance of existing regimens, it is imperative to explore novel, safe, cost-efficient adjuvant approaches. Such alternative interventions can broaden the available treatment options, optimize clinical outcomes, and alleviate the financial and psychological burdens of ART for women with diminished ovarian reserve. Therefore, further investigations into innovative adjuvant strategies are essential to improve pregnancy rates in POR populations (Zheng et al., 2025). (Giannelou et al., 2020; Jeve and Bhandari, 2016).

Acupuncture has been a controversial and debatable aspect of ART (Zhang et al., 2023; Hullender Rubin et al., 2018). Although the use of acupuncture has been studied as an adjunctive therapy to improve the level of fertility, most of the studies conducted are often constrained by small samples and absence of randomized controlled trials (RCTs) (Jang et al., 2020). Considering these difficulties, the proposed study attempts to evaluate the effectiveness of the acupuncture-assisted method of treatment in enhancing the IVF among the women with POR. In order to have more homogenous population of the study and to improve the relevancy and applicability of the findings, we selected patients in particular on the basis of the Bologna criteria (Cedars, 2022). These criteria are known as more suitable in fertility research to evaluate the therapeutic interventions in women with POR since they enable an assessment of ovarian reserve and other factors more standardized.

In our retrospective study, we analysed a large cohort of 2,133 women diagnosed with POR. Our findings indicate that acupuncture could be a helpful adjunctive therapy for enhancing both the quantity and quality of oocytes retrieved during IVF, as well as reduce cycle cancellation rates. These results align with the conclusions of our previous meta-analysis (Wang et al., 2023), which identified acupuncture as a promising treatment option for women with POR. Our study also provides additional support for the hypothesis that acupuncture may enhance folliculogenesis and improve oocyte competence. This has been believed to occur via mechanisms such as neuroendocrine control, which may aid in regulating hormones, and increased microcirculation, which enhances blood flow to the ovaries and may help optimise the ovarian environment to generate better oocytes. These mechanisms are believed to be synergistic in increasing the ovarian responses of women whose ovarian reserve is poor, leading to improved IVF outcomes (Feng et al., 2021). In analyzing the lack of a statistically significant difference in LBR, several factors warrant careful consideration. Despite the large sample size, the inherent heterogeneity of the POR population (e.g., varying etiologies and severities of diminished ovarian reserve) may have obscured a more subtle treatment effect on this final, clinically complex endpoint. Furthermore, while the follow-up was sufficient to capture live births, we cannot rule out that unmeasured confounding factors, such as variations in endometrial receptivity and embryo transfer techniques, which significantly impact LBR, were not fully balanced between groups (Neykova et al., 2022; Gerris, 2009). Although the acupuncture group showed higher CPR, these differences were not significant after adjustment, indicating the potential impact of these confounders. Furthermore, the inability to estimate these confounding influences and the variability in exposure limits the possibility of making causal inferences. These limitations emphasize the need for further studies with standardized treatment protocols, well-designed RCTs, and sufficient sample sizes to better determine acupuncture’s effectiveness for women with POR. Multicenter trials would also provide a more diverse and representative sample, helping to identify specific subpopulations of women most likely to benefit from acupuncture as part of their IVF treatment.

Dosage is a crucial factor influencing the effectiveness of acupuncture (Wang et al., 2024; Zhang et al., 2014). As our previous research has shown that the dosage of acupuncture is a key determinant of clinical outcomes in fertility treatments, directly impacting LBR (Zheng et al., 2025). Empirical data confirms this dose-response relationship: both 12 sessions and 6-8 sessions have been linked to significantly higher odds of live birth (Hullender Rubin et al., 2015; Hsu et al., 2017). This principle extends beyond reproductive medicine; managing chronic conditions like knee osteoarthritis and severe constipation typically requires a minimum of 10–28 sessions for substantial efficacy (Liu et al., 2017; Hershman et al., 2018). The profound influence of dosage underscores why LBR results vary across clinical trials. Based on this evidence, we conducted a subgroup analysis to investigate the effect of acupuncture dosage on fertility outcomes. The outcome of our analysis revealed that the positive impact of acupuncture on improving the number of obtained oocytes was not dependent on the clinical subgroups identified in the study, such as age or ovarian reserve at baseline. The result supports the fact that acupuncture can introduce significant advantages to POR women, independent of their factors. In addition, subgroup analyses were conducted based on patient age, pre-treatment AMH levels, and AFC, which are widely used in clinical practice to assess ovarian reserve and predict treatment response (Ferraretti et al., 2011; Grisendi et al., 2019). We used the values that are more likely to be diagnostic of POR as specific criteria for stratification, in order to more accurately analyze the treatment response in patients with different ovarian reserve levels (Hochberg et al., 2024). The results revealed that, the number of oocytes obtained in the acupuncture group was significantly higher than that in the control group. Future studies may consider developing tailored acupuncture treatment protocols for different types of POR populations and explore the optimal timing for acupuncture interventions.

This research has several limitations. First, it is a retrospective cohort study; thus, because it did not use randomised group assignment, there might have been a selection bias. Indicatively, there were chances that patients in the acupuncture group were more likely to seek adjunctive treatment or may have had varied baseline characteristics. Moreover, this was a single-centre study, and therefore, the results may not apply to the general population. To undertake such research studies, future multi-centre, prospective RCTs are necessary to enhance the applicability and stability of the conclusions.

## 5 Conclusion

These findings suggest that acupuncture could be a valuable adjunct to ART for POR women, particularly in women with specific reproductive characteristics, a higher dose of acupuncture appears to offer greater therapeutic benefit.
