# Supplementary material for: Association between acupuncture and IVF outcomes in women with poor ovarian response: a retrospective cohort study
Source: Front Cell Dev Biol. 2026 Jul 8;14:1756473. doi: 10.3389/fcell.2026.1756473 (PMC13389486; doi:10.3389/fcell.2026.1756473)
Supplement: Supplementary file 1 [file Table1.docx]

Supplementary Material

# Supplementary Tables

## Supplemental Table 1

| **Acupoints** | **Location** | **Manipulation** |
| --- | --- | --- |
| GV 20  (Baihui) | 5 cun* directly above the midpoint of the front hairline. | Transverse insertion to a depth of 0.5 cun–0.8 cun |
| CV12 (Zhongwan) | 4 cun to the navel, on the upper abdomen middle | Inserted vertically to a depth of 1.0 cun–1.5 cun |
| ST25 (Tianshu) | Bilateral, 2 cun lateral to the umbilicus | Inserted vertically to a depth of 1.0 cun–1.5 cun |
| GB26 (Daimai) | Bilateral, 1.8cun below to Zhangmen (Zhangmen,  below the free end of the 11th floating rib). | Inserted vertically to a depth of 1.0 cun–1.5 cun |
| CV 7  (Qihai) | 1.5 cun below the umbilicus, on the anterior midline | Inserted vertically to a depth of 1.0 cun–2.0 cun |
| CV4 (Guanyuan) | 3 cun below the umbilicus, on the anterior midline | Inserted vertically to a depth of 1.0 cun–2.0 cun |
| KI12  (Dahe) | Bilateral, 0.5 cun lateral to the lower anterior midline | Inserted vertically to a depth of 0.5 cun–1.0 cun |
| EX-CA1  (Zigong) | Bilateral, 4 cun lateral to the lower anterior midline | Oblique insertion to the uterus location direction  to a depth of 0.8 cun–1.2cun |
| SP10  (Xuehai) | Bilateral, 2 cun above the upper border of the patella | inserted vertically to a depth of 1.0 cun–1.5 cun |
| ST36  (Zusanli) | Bilateral, 3 cun directly below Dubi, and one  finger-breadth lateral to the anterior border of the tibia. (Dubi, in the lateral depression of the patellar ligament, when the knee is flexed) | Inserted vertically to a depth of 1.0 cun– 2.0 cun |
| SP6  (Sanyinjiao) | Bilateral, 3 cun above the tip of the medial malleolus. | Inserted vertically to a depth of 1.0 cun–1.5 cun |
| LR3  (Taichong) | Bilateral, in the depression anterior to the junction of  1st and 2nd metatarsal bones. | Inserted vertically to a depth of 0.5 cun– 0.8 cun |
| BL23  (Shenshu) | bilateral, 1.5 cun lateral to the depression below the  spinous process of the 2nd lumbar vertebra | Inserted vertically to a depth of 0.5 cun–1.0 cun |
| BL 32  (Ciliao) | Bilateral, in the sacral region, below the anterior  superior iliac spine, and precisely at the site of the second sacral foramen. | Inserted vertically to a depth of 1.0 cun–1.5 cun |
| KI 3  (Taixi) | Bilateral, on the medial side of the foot, at the  depression between the tip of the medial malleolus and the Achilles tendon | Inserted vertically to a depth of 0.5 cun–1.0 cun |

## Supplemental Table 2

STROBE Statement—checklist of items that should be included in reports of observational studies

|  | **Item No** | **Recommendation** | **Page  No** |
| --- | --- | --- | --- |
| **Title and abstract** | 1 | (*a*) Indicate the study’s design with a commonly used term in the title or the abstract | Page 1 |
|  |  | (*b*) Provide in the abstract an informative and balanced summary of what was done and what was found | Page 1-2 |
| **Introduction** | | | |
| Background/rationale | 2 | Explain the scientific background and rationale for the investigation being reported | Page 2-3 |
| Objectives | 3 | State specific objectives, including any prespecified hypotheses | Page 3 |
| **Methods** | | | |
| Study design | 4 | Present key elements of study design early in the paper | Page 3 |
| Setting | 5 | Describe the setting, locations, and relevant dates, including periods of recruitment, exposure, follow-up, and data collection | Page 3-4 |
| Participants | 6 | (*a*) *Cohort study*—Give the eligibility criteria, and the sources and methods of selection of participants. Describe methods of follow-up  *Case-control study*—Give the eligibility criteria, and the sources and methods of case ascertainment and control selection. Give the rationale for the choice of cases and controls  *Cross-sectional study*—Give the eligibility criteria, and the sources and methods of selection of participants | Page 3 |
|  |  | (*b*) *Cohort study*—For matched studies, give matching criteria and number of exposed and unexposed  *Case-control study*—For matched studies, give matching criteria and the number of controls per case | Page 4 |
| Variables | 7 | Clearly define all outcomes, exposures, predictors, potential confounders, and effect modifiers. Give diagnostic criteria, if applicable | Page 4 |
| Data sources/ measurement | 8* | For each variable of interest, give sources of data and details of methods of assessment (measurement). Describe comparability of assessment methods if there is more than one group | Page 4 |
| Bias | 9 | Describe any efforts to address potential sources of bias | Page 4 |
| Study size | 10 | Explain how the study size was arrived at | Page 4 |
| Quantitative variables | 11 | Explain how quantitative variables were handled in the analyses. If applicable, describe which groupings were chosen and why | Page 4 |
| Statistical methods | 12 | (*a*) Describe all statistical methods, including those used to control for confounding | Page 4 |
|  |  | (*b*) Describe any methods used to examine subgroups and interactions | Page 4 |
|  |  | (*c*) Explain how missing data were addressed | Page 4 |
|  |  | (*d*) *Cohort study*—If applicable, explain how loss to follow-up was addressed  *Case-control study*—If applicable, explain how matching of cases and controls was addressed  *Cross-sectional study*—If applicable, describe analytical methods taking account of sampling strategy | Page 4 |
|  |  | (*e*) Describe any sensitivity analyses | Page 4 |
